# Increased expression of claudin-17 promotes a malignant phenotype in hepatocyte via Tyk2/Stat3 signaling and is associated with poor prognosis in patients with hepatocellular carcinoma

**DOI:** 10.1186/s13000-018-0749-1

**Published:** 2018-09-15

**Authors:** Lemeng Sun, Liangshu Feng, Jiuwei Cui

**Affiliations:** 1 Stem Cell and Cancer Center, The First Bethune Hospital, Jilin University, Changchun, Jilin, 130021 People’s Republic of China; 2 Department of Neurology and Neuroscience Center, First Hospital of Jilin University, Changchun, Jilin, China

**Keywords:** Hepatocytes, Tight junction, Migration, CLDN17, Tyrosine kinase 2, Signal transducer and activator of transcription 3

## Abstract

**Background:**

Hepatocellular carcinoma (HCC) is the second leading cause of cancer death in Asia; however, the molecular mechanism in its tumorigenesis remains unclear. Abnormal expression of claudins (CLDNs), a family of tight junction (TJ) proteins, plays an important role in the metastatic phenotype of epithelial-derived tumors by affecting tight junction structure, function and related cellular signaling pathways. In a previous study, we used a tissue chip assay to identify CLDN17 as an upregulated gene in HCC. Here we aimed to use molecular biology technology to explore the effect of CLDN17 on the malignant phenotype of HCC and the underlying molecular mechanism, with the objective of identifying a new target for HCC treatment and the control of HCC metastasis.

**Method:**

The expression levels of CLDN17 in HCC tissues and histologically non-neoplastic hepatic tissues were explored by immunohistochemistry. Stable transfection of the hepatocyte line HL7702 with CLDN17 was detected by real-time polymerase chain reaction (PCR), western blotting and immunofluorescence. The impact of CLDN17 on the malignant phenotype of HL7702 cells in vitro was assessed by a Cell Counting Kit-8 (CCK8) assay, a Transwell assay and a wound-healing experiment. Western blotting was utilized to detect the activation state of Tyrosine kinase 2 (Tyk2) / signal transducer and activator of transcription3 (Stat3) pathway. A Tyk2 RNA interference (RNAi) was utilized to determine the impact of the Tyk2/Stat3 signaling pathway on the malignant phenotype of hepatocytes.

**Results:**

In this work, our research group first found that CLDN17 was highly expressed in HCC tissues and was associated with poor prognosis. In addition, we demonstrated that CLDN17 affected the Stat3 signaling pathway via Tyk2 and ultimately enhanced the migration ability of hepatocytes.

**Conclusion:**

In conclusion, we confirmed that the upregulated expression of CLDN17 significantly enhances the migration ability of hepatocytes in vitro and we found that the activation of the Stat3 pathway by Tyk2 may an important mechanism by which CLDN17 promotes aggressiveness in hepatocytes.

## Background

Previous studies have shown that the metastasis of epithelial-derived tumors is accompanied by abnormalities in tight junction (TJ) structure and function [1, 2]. Claudins (CLDNs) are the key proteins that form TJ, and accumulating evidence suggests that tumor cells frequently exhibit changes in the expression and localization of CLDNs [3]. For example, CLDN1 demonstrated to be overexpressed in colorectal cancer (CRC) compared with the level in the normal mucosa, and CLDN1 targeting with an anti-CLDN1 monoclonal antibody (mAb) resulted in decreased growth and survival of colorectal cancer (CRC) cells, suggesting that CLDN1 could be a new potential therapeutic target for CRC [4]. In addition, a database-augmented, exosome-based mass spectrometry approach identified circulating CLDN3 as a biomarker in patients with prostate cancer [5]. Moreover, high-level cytoplasmic CLDN3 expression is an independent predictor of poor survival in patients with breast cancer [6]. The TJ protein CLDN4 has been reported to be overexpressed in advanced ovarian cancer (OC) and Kaplan-Meier survival analyses and the log-rank test suggest that high expression of CLDN4 may have prognostic value in OC [7]. These observations revealed that the alterations in CLDNs expression may be related to tumorigenesis and cancer progression in various types of human carcinoma.

Additionally, CLDNs have been shown to participate in the transduction of intracellular/extracellular signals and may be related to tumorigenesis and cancer progression in human various carcinomas [8, 9]. For instance, genetic and pharmacological studies confirmed that the expression of CLDN3 was downregulated in colon cancer and that the loss of CLDN3 induced Wnt/β-catenin activation in a transducer and activator of transcription 3 (Stat3)-dependent manner to promote colon cancer malignancy [10]. Besides, a recent study revealed that enhanced CLDN18 expression activated ERK1/2 to contributed to the malignant potentials of bile duct cancer [11]. Our preliminary work showed that CLDN17 was strongly expressed in HCC tissues and cell lines and weakly expressed in non-neoplastic tissues and hepatocyte lines, which revealed that upregulated CLDN17 expression may play a role in the development of HCC. Furthermore, gene chip screening revealed that CLDN17 overexpression activated the tyrosine kinase 2 (Tyk2)/Stat3 pathway signaling pathway. To date, there has been no report on the impact of CLDN17 on the malignant phenotype of hepatocytes. In this study, we utilized molecular biology and other techniques to study the role and mechanisms of CLDN17 in malignant phenotype of hepatocytes and to identify novel targets for HCC treatment and the control of early metastasis.

## Methods

### Antibodies

Rabbit polyclonal antibodies against CLDN17 (cat. no. ab233333) and mouse anti-human β-actin (cat. no. ab8226) were purchased from Abcam (Massachusetts, US). Rabbit anti-human phospho-Stat1 (cat. no. #7649), rabbit anti-human phospho-Stat3 (cat. no. #9145, rabbit anti-human phospho-Tyk2 (cat. no. #68790), rabbit anti-human Stat1 (cat. no.#14,994), rabbit anti-human Stat3 (cat. no. #9139) and rabbit anti-human Tyk2 (cat. no. #13531) were purchased from Cell Signaling Technology (Boston, USA).

### Cell culture

Human hepatocyte line (HL7702) and HCC cell lines (HepG2, Hep3B and Huh1) utilized in this study were purchased from Shanghai Cell Bank of the Chinese Academy of Sciences. These cell lines were cultured in Dulbecco’s modified Eagle’s medium supplemented with 10% fetal bovine serum (FBS) at 37 °C in a humidified incubator containing 5% CO_2_.

### Plasmid construction and transfection

The plasmid p-EGFP-C1/CLDN17 (NM_012131) was constructed and amplified by KeyGen BioTech Company. Two micrograms of plasmid DNA was transfected into cells using the SuperFect Transfection Reagent (TaKaRa, Japan) according to the manufacturer’s protocol. A cell line stably expressing CLDN17 was selected in medium containing G418 (Thermo Fisher Scientific, Waltham, MA).

### Real-time polymerase chain reaction (PCR)

Total RNA was extracted using a Perfect Pure RNA Cultured Cell Kit (Thermo Fisher Scientific, Waltham, MA) according to the manufacturer’s protocol. Real-time PCR reactions was carried out as previously described [12]. The primer pairs used for CLDN17 and glyceraldehyde phosphate dehydrogenase (GAPDH) were as follows: CLDN17 forward (5′-ACCCAGCCATCCACATAG-3′) and reverse (5′- CCCTTGCTTCTTTCTGTTG-3′); and GAPDH forward (5’-AACGTGTCAGTCGTGGACCTG-3′) and reverse (5’-AGTGGGTGTCGCTGTFGAAGT-3′). The relative expression was based on the expression ratio of a target gene compared with that of GAPDH.

### Western blotting

A bicinchoninic acid (BCA) Protein Assay Kit (Pierce Chemical Co., Rockford, Illinois, USA) was utilized to detect protein concentrations. Total protein (30 micrograms) was separated via 10% sodium dodecyl sulfate-polyacrylamide gel electrophoresis (SDS-PAGE) gel and then transferred onto a nitrocellulose membrane (Millipore, Temecula, California, USA). Next, the membrane was blocked and investigated with the following primary antibodies: rabbit anti-human phospho-Stat1, rabbit anti-human Stat1, rabbit anti-human phospho-Stat3, rabbit anti-human Stat3, rabbit anti-human phospho-Tyk2, rabbit anti-human Tyk2, rabbit anti-human CLDN17 and mouse anti-human β-actin. After 3 washes with phosphate-buffered saline (PBS), the membrane was incubated with horseradish peroxidase (HRP)-conjugated secondary antibody (Santa Cruz Biotechnologies, California, USA) at a 1:1000 dilution at 4 °C. Immunoreactive bands were detected using ECL western blot reagents (GE, Fairfield, Connecticut, USA) and analyzed with Image Lab 6.0.1 Software from Bio-Rad Laboratories.

### Immunofluorescence method

The cells were fixed with 4% paraformaldehyde for 10 min at room temperature (RT) and then permeabilized with 0.1% Triton X-100 (Sigma-Aldrich, cat. no. 9002-93-1). Then, after blocking with 2% bovine serum albumin (Bote Biotechnological Corporation, Jilin, China) diluted in PBS for 30 min, the cells were probed with a primary rabbit anti-human CLDN17 antibody, which was diluted in blocking solution (1:1000 dilution) for 30 min at RT. The cells were incubated with Alexa Fluor®647-conjugated anti-rabbit IgG antibody (ab150093, Santa Cruz Biotechnologies, California, USA) at a 1:1000 dilution.

### Cell counting Kit-8 assay

Cell proliferation curve generated by the colorimetric water-soluble tetrazolium salt assay using a Cell Counting Kit-8 (Dojindo, Kumamoto, Japan) as the protocol. The cells were seeded into 96-well plates in triplicate, and cell proliferation was recorded per 12 h for 4 days.

### Wound-healing assay

The cells were maintained in a monolayer at 70% confluence on 24-well plastic dishes and the monolayer was scratched with a 100-μl pipette tip. The wounds were photographed light microscope (E100, Nikon Instruments Inc., Japan) (magnification × 200) at the same location at 0, 12 and 24 h.

### Transwell chamber method

The cells were grown in a monolayer at 90% convergence and were maintained in FBS-free medium for 12 h. Matrigel (BD Biosciences, cat. no. 356234) was added to the upper Boyden chamber (Millipore, Bedford, MA) in 24-well plates and the plates were maintained in a cell incubator at 37 °C for 15 min. Then, medium containing chemotactic factors, which had been collected from the cell culture, was added to the 24-well plate. The cells were supplemented with Matrigel and cultured in a cell incubator at 37 °C for 6 h.

### RNA interference (RNAi) method

Frozen glycerol bacterial stocks containing pGCSIL-scramble and pGCSIL-Tyk2-RNAi were purchased from Nanjing KeyGen Biotech Co., Ltd. The target was Tyk2-RNAi (29473), and the control insert sequence was pGCSIL-scramble. HEK 293 T cells (0.2 × 107) were seeded and maintained for 24 h to achieve 70–80% confluence in 6-well dishes (Costar, Cam- bridge, MA). Three plasmids, including of pGCSIL-Tyk2-RNAi or pGCSIL-scramble, 5 μg of the packaging vector pHelper 1.0 and 5 μg of a vesicular stomatitis virus glycoprotein (VSVG) expression plasmid vector, were added to Opti-MEM, with a final volume of 1.0 ml. Then, 50 μl of Lipofectamine was added to 950 μl of FBS-free medium. These two solutions were mixed and added to the cells. Lentiviral particles were harvested 48 h after transfection, and the viral titer was determined by counting green fluorescent protein (GFP)-expressing cells under a fluorescence microscopy (Nikon Diaphot 300®) with filters 96 h after transfection.

### Patients and tissue samples

Biopsies were collected from 52 patients with pathologically confirmed the diagnoses of HCC who received treatment at The First Bethune Hospital of Jilin University between June 2007 and May 2012. The patients were carefully chosen based on the following criteria: no history of radiotherapy or chemotherapy and no prior malignant disease. The grade and classification of the HCC patients were based on the American Joint Committee on Cancer (AJCC) tumor node metastasis (TNM) staging system. Thirty cases of histologically non-neoplastic hepatic tissues and 10 cases of cirrhosis tissues were also obtained from hepatitis B virus infected patients who were treated at The First Bethune Hospital, Jilin University during the period between October 2006 and September 2011 that were identified to be histologically non-neoplastic. There were 16 men and 14 women with an average age of 49 years. The medical records of the patients were reviewed to determine the clinical and pathological characteristics.

### Immunohistochemistry

An immunohistochemistry was utilized to explore the expression patterns of CLDN17 in 52 HCC tissues, 10 cirrhosis tissues and 30 non-neoplastic hepatic tissues. Of the 52 cases, 42 cases exhibited HBsAg infection, 17 cases exhibited occurrence and metastasis, and 34 cases were coupled with cirrhosis. The experimental method was described previously [13], and the antibody utilized was a rabbit anti-human CLDN17 antibody. The evaluation of protein expression levels was based on the percentage of positively stained tumor cells in combination with the staining intensity as previously described [14].

### Follow-up

The patients with a pathologically confirmed diagnosis of HCC were followed-up for 60 months after diagnosis to assess occurrence and metastasis and to determine survival. The survival status of the patients was determined through a telephone interview or an outpatient visit before December 2017.

### Statistical methods

All of the experiments were repeated 3 times, and all of the data are based on the mean ± SD of at least 3 experimental results. The experimental results were analyzed using Student’s t-test, and the prognostic significance and value of CLDN17 expression was determined by the Chi-square test/Chi-square goodness-of-fit test. *P* < 0.05 was considered statistical significance.

## Results

### The expression of CLDN17 was upregulated in HCC cell lines and tissues

**Fig. 1:**
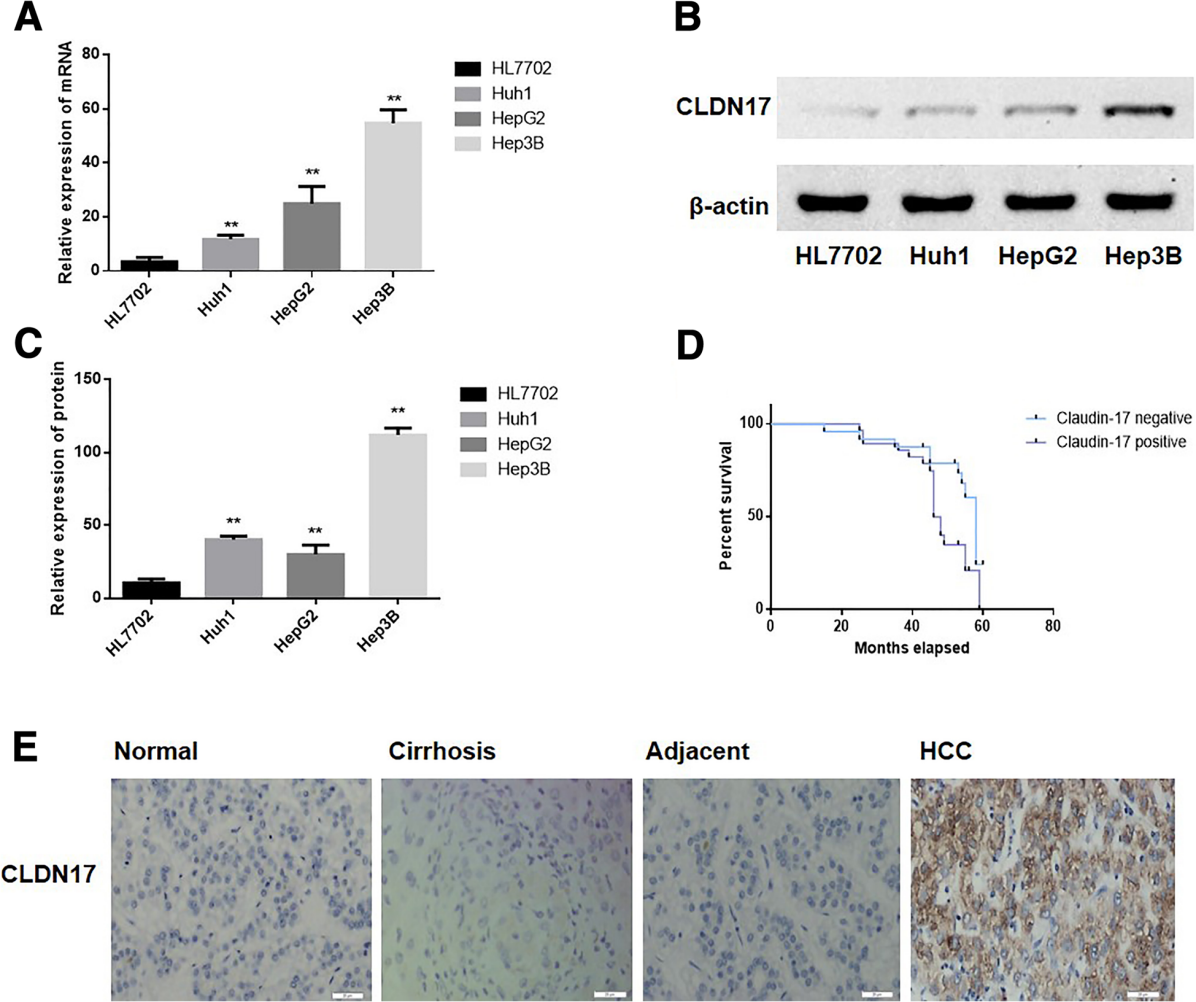
The expression levels of CLDN17 in HCC cell lines and tissues. **a** The relative mRNA level of CLDN17 in the hepatocyte line and HCC cell lines; (**b**) The relative protein expression of CLDN17 in the hepatocyte line and HCC cell lines; (**c**) The corresponding statistical analysis of protein expression in the hepatocyte line and HCC cell lines; (**d**) The correlation between the expression of CLDN17 and survival in HCC patients; (**e**) The protein expression of CLDN17 in hepatocyte tissues, cirrhosis tissues, tissues adjacent to the tumors and HCC tissues. Note: * represents *P* < 0.05, ** represents *P* < 0.01, compared with the empty vector groups

Real-time PCR and western blotting were utilized to detect the expression of CLDN17 in the human hepatocyte line and the HCC cell lines (Huh1, HepG2 and Hep3B). We found that the mRNA and protein expression levels of CLDN17 were low or absent in the human hepatocyte line HL7702 but high in HCC cell lines Huh1, HepG2 and Hep3B (Fig. 1a-c).

CLDN17 expression was explored in 52 HCC tissues, 10 histologically non-neoplastic cirrhosis tissues, 10 histologically non-neoplastic tissues adjacent to the tumor and 30 histologically non-neoplastic hepatic tissues. As shown in Fig. 1e, the expression of CLDN17 in hepatic tissues and HCC tissues is located mainly in the cytoplasm and membrane. High expression of CLDN17 was observed in 53.8% (28/52) of HCC tissues 30.0% (3/10) of non-neoplastic cirrhosis tissues, 40.0% (4/10) of histologically non-neoplastic tissues adjacent to the tumor and in 40.0% (12/30) of hepatic tissues (*P =* 0.0001 < 0.001) (Table 1).

**Table 1:** Expression of CLDN17 and the clinicopathological characteristics in HCC patients

Item	*n*	CLDN17 (+)	CLDN17 (−)	*P*
HCC tissues	52	28	24	< 0.01*
Hepatic tissues	30	12	18	
Age (years)				
≤ 60	21	11	10	1.000
> 60	31	17	14	
HbsAg				
+	42	23	19	1.000
-	10	5	5	
Cirrhosis				
+	34	18	16	1.000
-	18	10	8	
Occurrence and metastasis				
+	17	13	4	< 0.01*
-	35	15	20	
Serum AFP (ng/ml)				
< 400	22	13	9	0.637
> 400	30	15	15	
TNM stage (AJCC)				
I~II	27	17	10	< 0.01*
III~IV	25	11	14	
Histological grade				
Well-differentiated	24	15	9	< 0.01*
Moderately and poorly differentiated	28	13	15	

^*^Statistical significance was found with the Chi-square test/Chi-Square Goodness-of-Fit Test

The log-rank test was utilized to analyze correlations between CLDN17 and clinical survival. As shown in Fig. 1d, patients with positive expression of the CLDN17 protein in tumors (median survival, 46.15 months) had a notably shorter survival than those with negative expression of the CLDN17 protein (median survival, 57.79 months).

The relationships between CLDN17 and clinical pathological indicators were also analyzed, and the expression of CLDN17 was not associated with age (*P* = 1.000), HBsAg absent status (*P* = 1.000), cirrhosis (*P* = 1.000), serum AFP level (*P* = 0.637) of HCC patients or clinical staging (*P* = 1.000) of HCC patients.

However, CLDN17 expression was associated with HCC occurrence and metastasis (*P* = 0.001 < 0.01), histological grade (*P* = 0.001 < 0.01) and TNM stage (*P* = 0.001 < 0.01) (Table 1).

### Stable transfection of a hepatocyte line with CLDN17

**Fig. 2:**
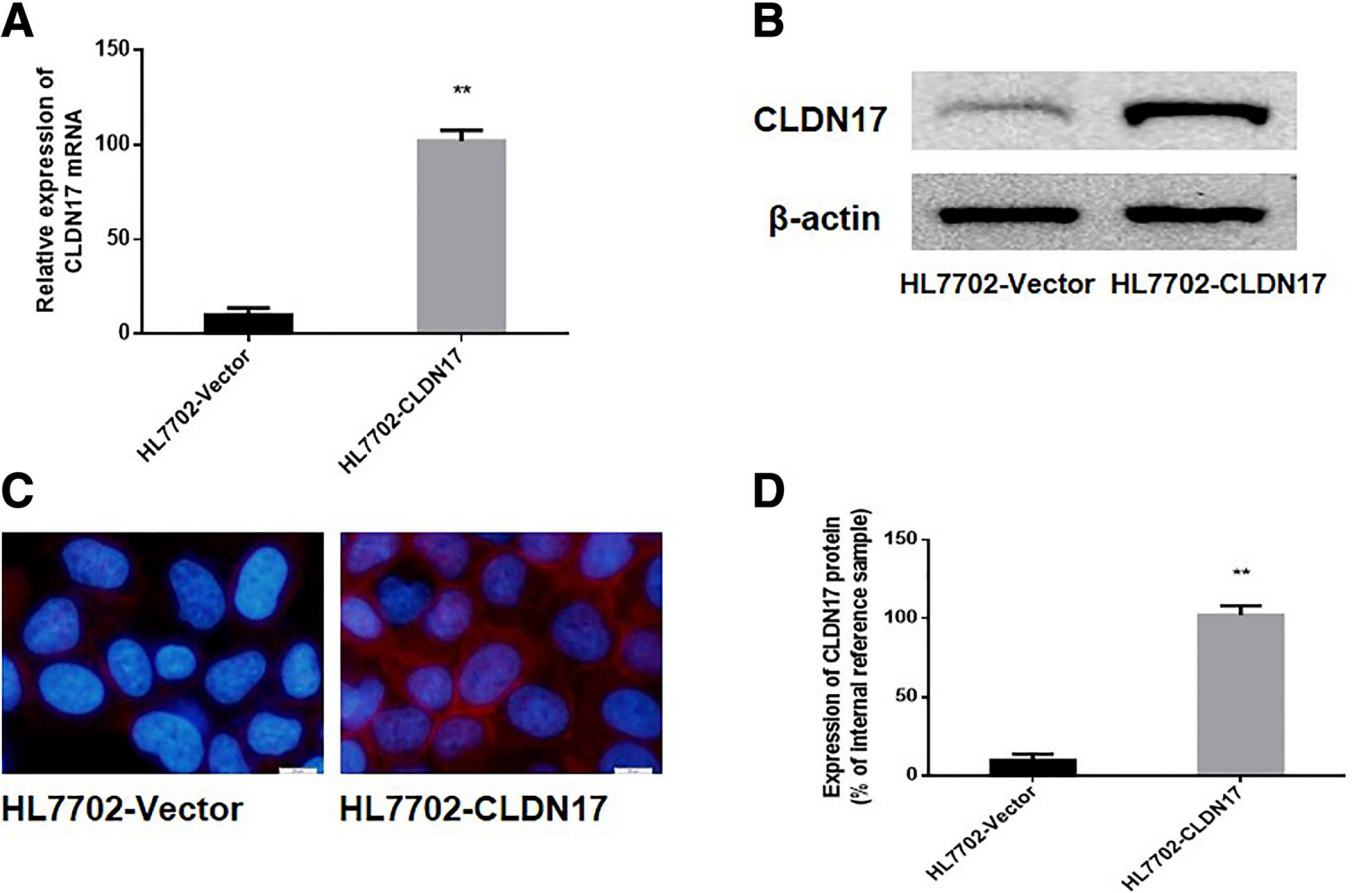
Characterization of the stable expression level of CLDN17. **a** Detection of CLDN17 in the HL7702 cell line by real-time PCR; (**b**) Detection of CLDN17 expression in the HL7702 cell line by western blotting; (**c**) Detection of CLDN17 expression in the HL7702 cell line by immunofluorescence; (**d**) The corresponding statistical analysis of protein expression in the HL7702 cell line. Note: * represents *P* < 0.05, and ** represents *P* < 0.01 compared with the empty vector groups

The p-EGFP-C1/CLDN17 plasmid was utilized to transfect HL7702 cells. After G418 screening, a monoclonal strain of HL7702 cells was obtained, which was termed HL7702-CLDN17. Real-time PCR and western blotting were also utilized to detect the expression of CLDN17 in cultured cells. The results showed that the mRNA and protein expression levels of CLDN17 in the HL7702-CLDN17 group were notably higher than those in the empty vector groups (*P =* 0.0001 < 0.01; *P =* 0.0001 < 0.01, respectively) (Fig. 2a, b and d). Immunofluorescence was utilized to detect the localization of CLDN17 in HL7702-CLDN17 cells. The results showed that the expression of CLDN17 was primarily localized to the cell cytoplasm and membrane (Fig. 2c). These results demonstrated that clonal HL7702 cell line that stably expressed CLDN17 had been successfully established.

### The impact of CLDN17 on the proliferation and migration of hepatocyte lines

**Fig. 3:**
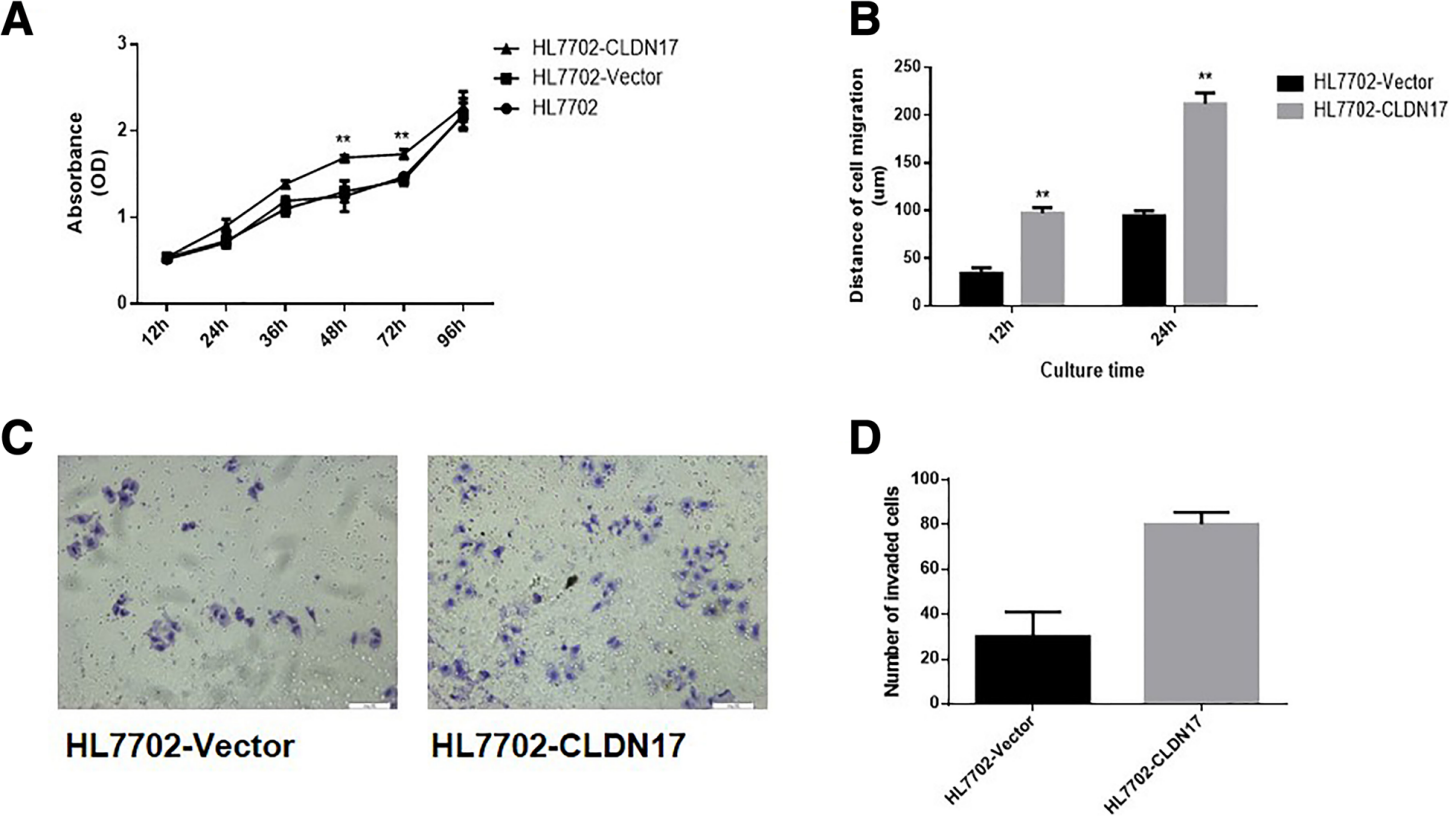
The impact of CLDN17 on the proliferation and migration ability of cells in vitro*.* **a** A growth curve for the HL7702 cell line was generated by the CCK*-8* method; (**b**) A wound healing assay was utilized to detect the migration ability of the HL7702 cell line in vitro; (**c**) The Transwell chamber method was utilized to detect the invasive ability of the HL7702 cell line in vitro; (**d**) The corresponding statistical analysis of invaded cells. Note: * represents *P* < 0.05 and ** represents *P* < 0.01 compared with the empty vector groups

The growth curve for the HL7702 cell line was generated by the CCK-8 method. The data revealed that the proliferation rates of HL7702-CLDN17 cells at 48 and 72 h were notably higher than those of the empty vector groups (Fig. 3a). A wound-healing experiment was utilized to detect the impact of CLDN17 on the migration ability of hepatocytes (Fig. 3b). The results showed that the migration distances of HL7702-CLDN17 cells were substantially greater than those of the empty vector groups at 12 and 24 h (*P =* 0.0022, < 0.01). The Transwell chamber method was also utilized to detect the migration ability of hepatocytes. Twelve hours after the cells were seeded, the cells that invaded through the membrane of the chamber were observed. The results showed that the number of invasive cells in the HL7702-CLDN17 group was notably higher than in the empty vector groups (Fig. 3c and d). These results suggested that CLDN17 clearly promoted the proliferation and migration ability of hepatocytes in vitro.

### The impact of CLDN17 on the Tyk2/Stat3 signaling pathway in hepatocytes

**Fig. 4:**
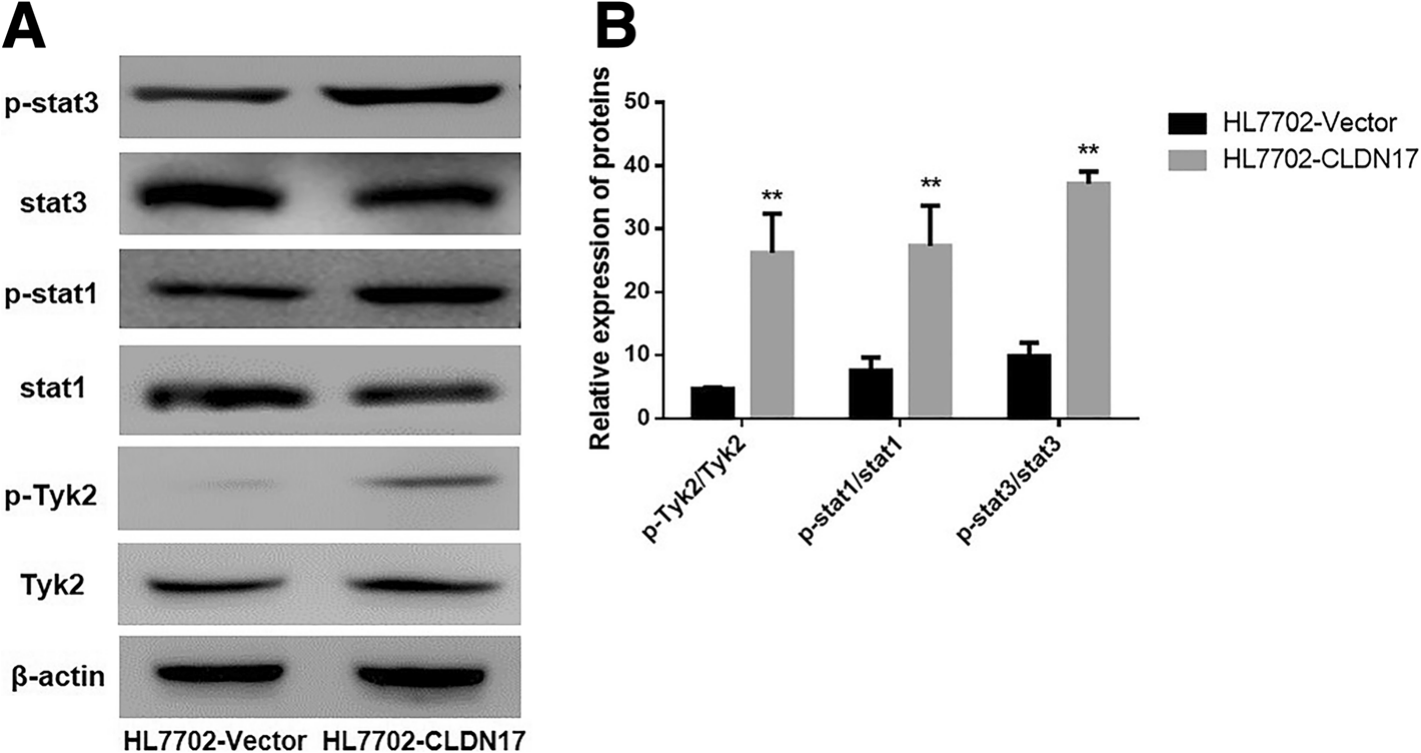
The impact of CLDN17 on the Tyk2/Stat3 signaling pathway. **a** Western blotting was utilized to detect the activation of the Stat3 signaling pathway in the HL7702 cell line; (**b**) The corresponding statistical analysis of the activation status of various Stat3 pathway components. Note: * represents *P* < 0.05 and ** represents *P* < 0.01 compared with the empty vector groups

Western blotting was utilized to explore the activation state of the Tyk2/Stat3 pathway. The results showed that after the overexpression of CLDN17, the phosphorylation levels of Stat1, Stat3 and Tyk2 were substantially increased in the HL7702 cells (Fig. 4a and b). These data suggested that CLDN17 upregulation notably enhanced the activation of the Tyk2/Stat3 signaling pathway in the HL7702 cell line.

### The impact of Tyk2/Stat3 signaling pathway activation on the invasion and migration ability of hepatocytes

**Fig. 5:**
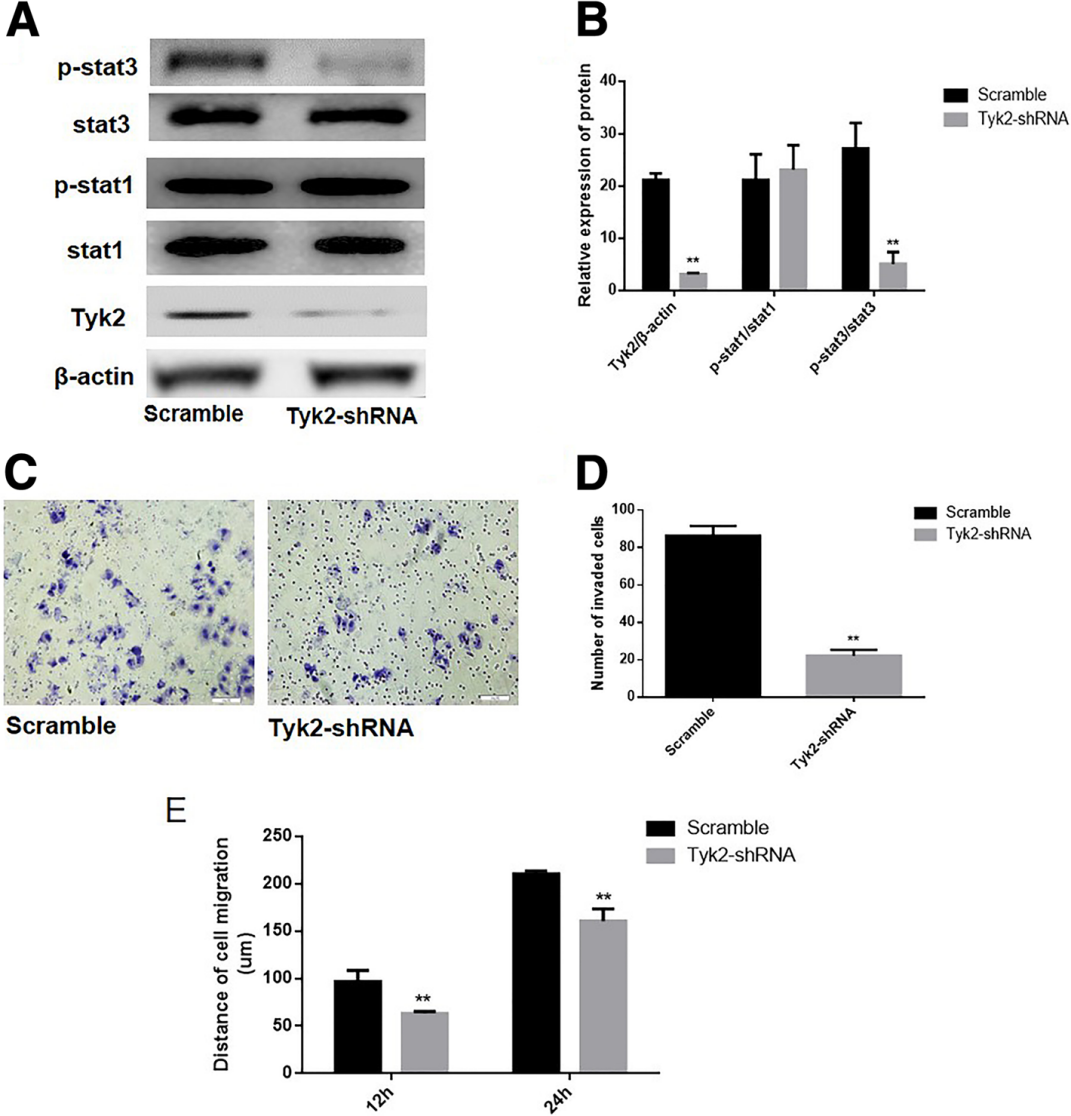
RNAi was utilized to silence Tyk2 expression in CLDN17-expressing cells. **a** Western blotting was utilized to examine the effects of silencing Tyk2 and activating the Stat3 signaling pathway in the HL7702 cell line; (**b**) The corresponding statistical analysis of the activation of the Stat3 signaling pathway; (**c**) The Transwell chamber method was utilized to detect the impact of Tyk2 silencing on the invasive ability of the cells in vitro; (**d**) The corresponding statistical analysis of invaded cells; (**e**) A wound healing assay was utilized to detect the migration ability of the HL7702 cell line in vitro. Note: * represents *P* < 0.05 and ** represents *P* < 0.01 compared with the scramble group

To determine the impact of the Tyk2/Stat3 signaling pathway on the invasion and migration ability of hepatocytes, the pGCSIL-scramble plasmid and the pGCSIL-Tyk2-RNAi plasmid were utilized to transfect HL7702-CLDN17 cells. Western blotting was utilized to analyze Tyk2 expression and the phosphorylation level of Stat1 and Stat3 in these cells, and the results showed that the protein expression of Tyk2 was markedly downregulated in Tyk2-RNAi cells compared with the scramble group (Fig. 5a and b). The data showed that after the knockdown of Tyk2, the phosphorylation level of Stat3 was notably downregulated while there was no significant change in the phosphorylation level of Stat1 in the HL7702 cell line that overexpressed CLDN17 (Fig. 5a).

A Transwell chamber assay and wound-healing assay were utilized to analyze the effect of Tyk2 on the invasive and migration ability of the examined cells. The results showed that the number of invasive HL7702 cells in the CLDN17-expressing cells was notably decreased following Tyk2 silencing in CLDN17-expressing cells (Fig. 5c and d). The migration distances of the Tyk2-RNAi cells were substantially shorter than those of the scramble group at 12 and 24 h (Fig. 5e).

## Discussion

A number of studies have focused on the role of CLDNs in the tumorigenesis of HCC. For instance, CLDN1 has been described as a key factor in the entry of hepatitis C virus (HCV) into hepatocytes, and upregulated expression of CLDN1 was revealed to contribute to the promotion of epithelial mesenchymal transition (EMT) via the c-Abl/Raf/Ras/ERK signaling pathway [15, 16]. Furthermore, it was demonstrated that CLDN3 is an epigenetically silenced tumor suppressor gene in HCC, and its overexpression notably inhibits metastasis by suppressing the EMT via the Wnt/β-catenin signaling pathway in HCC cells [13]. Besides, CLDN14 was epigenetically silenced via the trimethylation of lysine 27 on histone H3 (H3K27ME3) and was a novel prognostic biomarker in HCC [17]. Given the correlation between the expression levels of these CLDNs and the tumorigenesis of HCC, CLDNs represent potential novel therapeutic targets in patients with HCC.

CLDN17 is one of 27 members of the CLDN protein family, and our current understanding of the biological functions of CLDN17 is primarily limited to epithelial and epidermal permeability, barrier protection, and cell connections; reports on the relationship between CLDN17 and tumors are rare [18]. Our research group first found that CLDN17 expression was highly expressed in HCC tissues, and we speculated that the high expression of this gene may be involved with the tumorigenesis and progression in patients with HCC. Moreover, in the present study, we confirmed that CLDN17 markedly promotes the invasive ability of the hepatocyte line HL7702. Similar to our study, several studies have identified specific CLDNs as pro-oncogenes in human various cancers. For instance, previous work has shown that CLDN1 plays a key role in inflammation-induced growth and progression in patients with colorectal carcinoma [19]. Furthermore, Philip, R. et al. reported that CLDN7 expression in colorectal cancer contributes to motility and invasion by promoting a shift towards EMT by recruiting EpCAM towards TACE/presenilin2 [20]. It was also revealed that CLDN7 is frequently overexpressed and promotes invasion in ovarian cancer [21]. However, in contrast to our results, other studies have shown that some CLDNs could be identified as tumor suppressor gene [22, 23]. For instance, the expression of CLDN1 was reduced in stage II and III rectal cancer and was established as a factor that correlates clearly with recurrence and poor prognosis [24]. In addition, the expression of CLDN6 was demonstrated to be silenced in cervical carcinoma tissues, and the restoration of CLDN6 expression suppressed cell proliferation and colony formation in cervical carcinoma cells in vitro, and tumor growth in vivo [25]. One potential reason for this difference is that the functions of CLDNs may be specific and dependent on different interacting molecules in different cells [26, 27]. In this manner, specific CLDNs may have specific impacts on the biological behavior of a given tumor [28–30].

At present, our results first indicated that the CLDN17 was overexpressed and highly associated with metastatic progression and prognosis in patients with HCC. Moreover, the overexpression of CLDN17 markedly promoted the invasion and migration abilities of the hepatocyte line HL7702. Furthermore, we also performed an initial exploration of the molecular mechanism associated with this effect, and we found that CLDN17 upregulation affected the Stat3 signaling pathway via Tyk2 and ultimately enhanced the migration ability of hepatocytes. Considering the limited therapeutic options for patients with HCC, the role of CLDN17 as a therapeutic target merits further exploration.

## Conclusion

There have been few reports on the roles of CLDN17 in tumors, and many problems related to the specific molecular mechanisms must still be researched. In the present study, we confirmed that the overexpression of CLDN17 notably enhanced the malignant phenotype of the hepatocytes. In addition, the induction of Tyk2/Stat3 signaling may be one of the most important mechanisms by which CLDN17 promotes the migration ability in hepatocytes.

## Abbreviations

AJCC: American Joint Committee on Cancer
CCK8: Cell Counting Kit-8
CLDNs: Claudins
FBS: Fetal bovine serum
GFP: Green fluorescent protein
H3K27ME3: Trimethylation of lysine 27 on histone H3
HCC: Hepatocellular carcinoma
HCV: Hepatitis C virus
Stat3: Signal transducer and activator of transcription3
TJ: Tight junction
TNM: Tumor node metastasis
Tyk2: Tyrosine kinase 2
